# On the influence of provenance to soil quality enhanced stress reaction of young beech trees to summer drought

**DOI:** 10.1002/ece3.2472

**Published:** 2016-10-21

**Authors:** Constanze Buhk, Marcel Kämmer, Carl Beierkuhnlein, Anke Jentsch, Jürgen Kreyling, Hermann F. Jungkunst

**Affiliations:** ^1^ Institute of Environmental Sciences, GeoecologyUniversity of Koblenz‐LandauLandauGermany; ^2^ BiogeographyUniversity of BayreuthBayreuthGermany; ^3^ Disturbance EcologyUniversity of BayreuthBayreuthGermany; ^4^ Institute for Botany and Landscape Ecology, Experimental Plant EcologyErnst Moritz Arndt University GreifswaldGreifswaldGermany

**Keywords:** climate change, drought adaptation, *Fagus sylvatica*, foliar δ^13^C, natural stable isotope signature, plasticity

## Abstract

Climate projections propose that drought stress will become challenging for establishing trees. The magnitude of stress is dependent on tree species, provenance, and most likely also highly influenced by soil quality. European Beech (*Fagus sylvatica*) is of major ecological and economical importance in Central European forests. The species has an especially wide physiological and ecological amplitude enabling growth under various soil conditions within its distribution area in Central Europe. We studied the effects of extreme drought on beech saplings (second year) of four climatically distinct provenances growing on different soils (sandy loam and loamy sand) in a full factorial pot experiment. Foliar δ^13^C, δ^15^N, C, and N as well as above‐ and belowground growth parameters served as measures for stress level and plant growth. Low‐quality soil enhanced the effect of drought compared with qualitatively better soil for the above‐ and belowground growth parameters, but foliar δ^13^C values revealed that plant stress was still remarkable in loamy soil. For beeches of one provenance, negative sandy soil effects were clearly smaller than for the others, whereas for another provenance drought effects in sandy soil were sometimes fatal. Foliar δ^15^N was correlated with plant size during the experiment. Plasticity of beech provenances in their reaction to drought versus control conditions varied clearly. Although a general trend of declining growth under control or drought conditions in sandy soil was found compared to loamy soil, the magnitude of the effect of soil quality was highly provenance specific. Provenances seemed to show adaptations not only to drought but also to soil quality. Accordingly, scientists should integrate information about climatic pre‐adaptation and soil quality within the home range of populations for species distribution modeling and foresters should evaluate soil quality and climatic parameters when choosing donor populations for reforestation projects.

## 1 Introduction

Drought stress of plants will most likely become a common phenomenon in the course of climate change (Hewitson et al., 2014). During severe droughts, even adult trees can suffer (Bréda, Huc, Granier, & Dreyer, 2006) but young progeny are especially prone to severe drought stress. Tree establishment is the most serious bottleneck for the regeneration of European beech (*Fagus sylvatica*) forests subject to drought (Gallé & Feller, 2007) as the root system of young trees is not sufficiently deep to access further water sources (Bréda et al., 2006). In particular, if the water retention capacity of the soil is low (e.g., in sandy soils), extreme drought events can have dramatic effects (Bréda et al., 2006; Geßler et al., 2007). Most natural ecosystems in Central Europe would be dominated by European beech (Leuschner, Meier, & Hertel, 2006); it is therefore both ecologically and economically highly relevant how this tree species will react to drought during all stages of its life cycle. *Fagus sylvativa* is especially sensitive to drought (Fotelli et al., 2009; Geßler, Keitel, Nahm, & Rennenberg, 2004; Robson, Sánchez‐Gómez, Cano, & Aranda, 2012): Early season (May–July) water supply is a crucial driver of beech growth (Scharnweber et al., 2011) and partially determines *F. sylvatica* distribution limits (Czúcz, Gálhidy, & Mátyás, 2011). The results of many studies (Czajkowski & Bolte, 2006; Madsen, 1995; Nielsen & Jørgensen, 2003; Tognetti, Johnson, & Michelozzi, 1995) that focus on drought reaction of beech vary from little effect (Leuzinger, Zotz, Asshoff, & Körner, 2005) to 25% mortality (Thiel et al., 2014) depending on site conditions or experimental setup. Previous studies have investigated drought effects on seedlings (Peuke & Rennenberg, 2011; Rose, Leuschner, Köckemann, & Buschmann, 2009), saplings (Gallé & Feller, 2007; Robson et al., 2012) and adult beech trees (Leuzinger et al., 2005; Nahm, Matzarakis, Rennenberg, & Geßler, 2007) of different provenances. Previous studies carried out their research on calcareous soils (Gärtner et al., 2008), organic soil (van Hees, 1997) or sand (Czajkowski & Bolte, 2006) and with or without fertilization (Harter et al., 2015 and Sánchez‐Gómez, Robson, Gascó, Gil‐Pelegrin, & Aranda, 2013; respectively), but the nutrient availability or soil quality was not focused at within the same experiment to highlight differences of beech growth on different soils. Whereas there is evidence that climate within the home range of a beech provenance determines the ability to cope with severe drought events (Nielsen & Jørgensen, 2003; Peuke, Schraml, Hartung, & Rennenberg, 2002; Rose et al., 2009; Tognetti et al., 1995), adaptation to specific soils and its interaction with provenance has not yet been studied systematically.

Typical response parameters to study the reaction of beech trees to drought are physiological characteristics such as the predawn water potential or gas exchange (Aranda, Gil, & Pardos, 2005; Tognetti et al., 1995), or morphological changes such as height increment, leaf area or root mass (Meier & Leuschner, 2008a,b; Rose et al., 2009; van Hees, 1997). Stable isotope signals (δ^13^C) have been used successfully to demonstrate the effect of drought stress (Fotelli, Rennenberg, Holst, Mayer, & Gessler, 2003; Geßler et al., 2004; Robson et al., 2012; Rose et al., 2009): The photosynthetic pathway usually discriminates against ^13^C isotopes because they are heavier and consequently diffuse more slowly into the plant. Under drought, however, stomata are kept closed when the water deficit becomes strong, so ^13^C isotopes inside the stomatal cavities are used for photosynthesis and discrimination values are lowered (Fotelli et al., 2003). In contrast to δ^13^C, it is not known whether δ^15^N in leaves of beech differ under drought (Peuke, Gessler, & Rennenberg, 2006), although such effects were observed in barley (Robinson et al., 2000). However, δ^15^N signals are known to be very provenance specific, reflect the pathway of N uptake and transport, and may be coupled with growth (Peuke et al., 2006).

To our knowledge, there are no experimental studies using stable isotope signals coupled with above and below ground biomass data to determine whether lower soil quality enhances drought reaction independently of provenance. Soil conditions may mitigate or exacerbate provenance‐specific reactions to severe drought as a result of possible pre‐adaptation to drought events in their home range coupled with the local soil conditions.

We expected that different provenances would show different stress levels under drought (mirrored in the foliar δ^13^C values) and that poorer sandy soil would enhance the stress reaction because water and nutrient shortage is more pronounced compared to loamy soils. We hypothesized that the effect of soil quality (soil texture and nutrient content) under drought differs among provenances in terms of growth (root and shoot biomass), nutrient supply (leaf carbon and nitrogen), and stress level (natural isotope signatures). We discuss these findings on the basis of the provenance‐specific climatic and soil conditions in the trees' home ranges. We expected that saplings originating from humid sites would suffer more from the drought treatment than saplings from Mediterranean sites. We also expected that adaptation to soil conditions in the area of origin would affect the magnitude of the stress reaction—whereby trees growing in soils similar to their area of origin would fare better under drought than individuals grown in different soil conditions.

## 2 Materials and Methods

### 2.1 Experiment—background

In the Event 3‐Landau experiment, a subproject of the Event Experiment series in Bayreuth (see e.g., Backhaus et al., 2014; Beierkuhnlein, Thiel, Jentsch, Willner, & Kreyling, 2011; Kreyling et al., 2011, 2012), two soils of differing quality were used and two‐year old saplings of different European beech provenances were tested on their reaction to severe early season drought (Thiel et al., 2014). Testing beech saplings in the second year after planting them in different mineral soils was especially relevant, as beech seedlings that establish in organic horizons start to penetrate the mineral soil properly in their second year and therefore soil characteristics start to matter. Here, we compare the foliar C, N, δ^13^C, and δ^15^N values under drought or control conditions on different soils with initial size, growth parameters during the experiment as well as above and below ground dry mass and tree survival 1 year after the treatment for beeches from Kempten, Hengstberg, Johanniskreuz (Germany), and Montejo de la Sierra (Spain) representing four of the six provenances studied by Thiel et al. (2014).

### 2.2 Experimental site

The Landau experimental site is located close to the Campus Landau of the University of Koblenz‐Landau, at the Julius Kühn‐Institute (JKI), Federal Research Centre for Cultivated Plants, Siebeldingen (49°13′03″N, 8°02′47″E, 202 a.s.l.). Mean annual temperature is 10.2°C and mean annual precipitation is 643 mm, distributed bimodally with a peak in May/June and another in November/December (data: German Weather Service).

### 2.3 Plant material and potting

Seedlings from different provenances of beech (*F. sylvatica*) were raised at the Bavarian Institute for Forest Seeding and Planting (ASP) in Teisendorf, Germany, in spring 2010. The beeches of this work originate from four different sites, summarized in Table 1. Soil characteristics within the home range were derived from regional soil maps and published studies. Soil texture classified according to the World Reference Base for Soil Resources 2014 (IUSS Working Group WRB, 2014). The summer heat moisture index (SHMI) was calculated as (mean temperature of warmest month)/(mean annual summer precipitation/1,000) according to Wang, Hamann, Spittlehouse, and Aitken (2006) using data derived from WorldClim (Hijmans, Cameron, Parra, Jones, & Jarvis, 2005). The sites Kempten (47°44′48″N 10°08′54″E) and Montejo de la Sierra (41°07′12″N 03°30′36″W) represent the extremes of SHMI, and the sites Hengstberg (50°08′00″N 12°11′00″E) and Johanniskreuz (49°18′14″N 07°50′07″E) are located in between with similar SHMIs. The latter provenances differentiate as beeches in Johanniskreuz stock on very poor and sandy soils while beeches in Hengstberg stock on more favorable loamy substrate (Table 1).

**Table 1:** Environmental characteristics of the home ranges of the beech provenances studied

Provenance	Region	Altitude (m.a.s.l.)	Soil origin and texture	SHMI^a^
Kempten, Germany	Alpine upland	803	Soil on Marl (Molasse), texture:loam (clay/silt/sand: 15/40/45)^b^	26
Hengstberg, Germany	Low mountain range Fichtelgebirge	569	Soil on Paleolithic granite rock, texture: clay loam (clay/silt/sand: 33/42/25)^c^	47
Johanniskreuz, Germany	Low mountain range Palatinate Forest	570	Soil on Mesozoic Buntsandstein, texture: sand to loamy sand (clay/silt/sand: <5/10/85–90)^d^	42
Montejo de la Sierra, Spain	High mountain range Sistema Central	1350	Soil on micaceous gneiss rock, texture: sandy loam (clay/silt/sand:14/16/70)^e^	80

a SHMI: summer heat moisture index (Wang et al., 2006).

b According to profile 21 in Jerz (1973). Soil texture was translated to international standard using the world reference base for soil resources (IUSS Working Group WRB, 2014).

c Signature G1 according to Geological Map 5838/5839 Selb/Schönberg (Mielke & Stettner, 1984). Detailed size classes were taken from Spielvogel, Knicker, and Kögel‐Knabner (2004) who studied texture of soils on similar substrates nearby (sample 13/G2).

d Data put to our disposal by the Forschungsanstalt für Waldökologie und Forstwirtschaft—Forstliches Umweltmonitoring.

e Data taken from Pardo, Gil, and Pardos (1997). Specified percentages of soil fractions were recalculated to 100% fine soil.

Beech saplings overwintered in wooden boxes covered with blankets in the Bayreuth Botanical Garden. Rootstocks were protected against damage and drying with a biodegradable wrap, which served after harvest to separate newly grown roots from old roots. The beech trees were planted into 12‐L plastic pots on 14 March 2011, and pots were placed on plant saucers to avoid water loss after watering.

Beech saplings were randomly chosen for each provenance and planted in sandy loam (henceforth loamy soil) or loamy sand (henceforth sandy soil). The loamy soil was a mixed sample of top soil of two different forests collected in the vicinity of Bayreuth. Laser analyses (Mastersizer, Malvern Instrument, University of Bayreuth) characterized the sandy loam as containing c. 68% sand, 21% silt, and 11% clay. The soil was sieved through a 1‐cm grid to homogenize it prior to potting. The sandy soil was created by adding 50% quartz sand from a local sand pit to the first soil, and the mixture was homogenized as above. Soil chemical analyses were carried out at the University of Bayreuth, Bayceer Centre, and pH and electric conductivity were measured at the University of Landau, Geoecology laboratory (Table 2).

**Table 2:** Soil nutrient characteristics of the two soils used in the experiment

	K (mg/kg)	Mg (mg/kg)	P (mg/kg)	NO_3_ (mg/kg)	NH_4_ (mg/kg)	N (%)	C_org_ (%)	pH (H_2_O)	EC (μs/cm)
Sandy loam	118	267	48.3	31	3.98	0.14	1.92	6.68	140
Loamy sand	54.8	1211	11.1	14.1	2.02	<0.1	0.56	7.16	117

### 2.4 Experimental setup

A fully crossed three‐factorial design was established including four different provenances, a drought versus control treatment, and sandy versus loamy soil with nine replicates per treatment. Pots were placed completely randomly outdoors at the JKI in Siebeldingen, close to Landau (49°13′03″N, 8°02′47″E). For the C, N, and isotope analyses, only four replicates of each group were randomly chosen and analyzed (64 beech samples in total). The plants were exposed to ambient precipitation and were additionally watered with groundwater if necessary to allow good establishment in the pots. On 13th April, a rainout shelter and a shading canvas were installed (for details, see Thiel et al., 2014). From 2nd May onwards, all plants received the 40 year average precipitation amount divided into two doses per week. For the drought treatment, no watering took place for a period of 36 days starting on 9th May ending on 13th June. The criterion to stop the drought treatment was that 20% of the individuals showed strong drought damage. During the drought treatment, the control pots were continuously watered according to the respective week's 40 years average. About 12 days after the start of the drought treatment, soil moisture in the sandy soil had dropped below the wilting point (pF = 4.2), approximately 1 week later this happened in the loamy soil (see figure 2 in Thiel et al., 2014).

### 2.5 Response parameters

Tree height and stem diameter were measured shortly after planting (19th March 2011), at the beginning of the drought (8th May) and after the drought treatment (14th June). Leaves were counted on 10th May and 15th June. Final tree height, aboveground dry mass (g), and root dry mass grown during the time of the experiment (g) were measured 1 year later on 10th April 2012 and the survival of the saplings was documented. To measure the root mass that had been produced since the potting of the saplings in the mineral soil, the roots that grew outside the biodegradable wrap were cut, dried, and weighed. Fine roots <1 mm diameter were separated and weight in addition to the total root dry mass.

Leaf samples for the determination of foliar C, N, δ^13^C, and δ^15^N were taken at the start of the drought treatment on 9th May and at the end of the drought period on 14th June. One medium‐sized leaf was taken from the upper part of the crown so as not to damage the tree even more after drought. The leaves were dried in paper bags for 3 days (60°C) immediately after sampling. After drying, each sample was ground into a homogenous fine powder using a ball mill with two sodium oxide balls for at least five minutes with 60 shakes per second. One to 2 ml of the ground material was transferred into tin capsules and analyzed at the Centre for Stable Isotope Research and Analysis in Göttingen, Germany, using an Elementary Analyzer NA 2500 (CE‐Instruments, Rodano, Milano, Italy) coupled to an isotope ratio mass spectrometer (Delta plus, Finnigan MAT, Bremen, Germany) through a Conflo III interface (Thermo Electron Coopertion, Bremen, Germany). δ^13^C values are expressed relative to the Vienna‐PDB standard, whereas δ^15^N values are expressed relative to the international standard (atmospheric nitrogen).

### 2.6 Statistical analyses

Linear models (LMs) were used to determine treatment effects on the change of foliar δ^13^C, δ^15^N, C, and N during the drought period, as well as on the final root mass, fine root mass to root mass ratio, aboveground biomass, and final tree height after 1 year. Explanatory factors included in the model were provenance, soil, and drought treatment as well as all interactions. To compare the provenance‐specific effects on drought in detail on the different soils (which was the main focus of the study), LMs were repeated separately for the two soil qualities as the complete model was too weak to uncover soil specific reactions of different provenances to drought.

Growth parameters and foliar δ^13^C, δ^15^N, C, and N before the drought treatment were analyzed with LMs including provenance, soil, and their interaction as explanatory factors. As all specimens had been treated equally before the drought treatment in May, eight replicates each were included in the analyses of foliar δ^13^C, δ^15^N, C, and 18 replicates each for the initial morphological characteristics.

One sample from Hengstberg had to be excluded from the leaf chemical analyses, as the values for C, N, and isotope signatures were out of the plausible range and a measurement error was suspected. Data were not normally distributed nor homoscedastic and were consequently rank‐transformed prior to analyses.

To test whether strong growth during the first year was correlated with higher foliar δ^15^N values, a variant of the LM was calculated adding “plant height in May” as a covariate. In addition, Spearman Rho correlation coefficients were calculated between tree height (May) or stem diameter (May) with foliar δ^15^N (May) and between the change in tree height and stem diameter change with the change in foliar δ^15^N over the course of the experiment.

## 3 Results

### 3.1 Changes of leaf parameters during the drought treatment

Overall, the full models provided a good fit for δ^13^C (*R* ^2^ = .75) and C (*R* ^2^ = .62) whereas N (*R* ^2^ = .32) was poorly explained (Table 3). Although several of the main factors explained a significant proportion of the variation in foliar δ^13^C, δ^15^N, C, and N, interactions between provenance × soil, provenance × drought, and soil × drought were only significant for foliar C (Table 3).

**Table 3:** Linear model's *F*‐ and *p*‐values on changes of foliar δ^13^C, δ^15^N, C, and N during the drought including all main effects (provenance, soil, and drought treatment) and all interactions (provenance × soil, provenance × drought, soil × drought, and provenance × soil × drought) as predictors

	Prov*F*/*p*	Soil*F*/*p*	Drought*F*/*p*	P × S*F*/*p*	P × D*F*/*p*	S × D*F*/*p*	P × S × D*F*/*p*	*R*²
δ^13^C change	4.22/**.01**	3.27/.077	125.11/**<.001**	0.33/.81	0.94/.43	0.39/.54	0.41/.74	.75
δ^15^N change	3.49/**.02**	10.58/**.002**	16.52**/<.001**	0.02/1	0.63/.6	0.13/.72	1.11/.36	.48
C (%) change	1.77/.17	24.83**/<.001**	0.89/.35	4.31/**.01**	8.54/**<.001**	9.14/**.004**	0.16/.92	.62
N (%) change	0.69/.56	4.9/**.032**	12.03/**.001**	0.86/.47	0.03/.99	0.12/.73	0.45/.72	.32

*p*‐Values indicating significant differences (*p* < .05) are written in bold.

#### 3.1.1 Changes in δ^13^C values during drought

**Figure 1:**
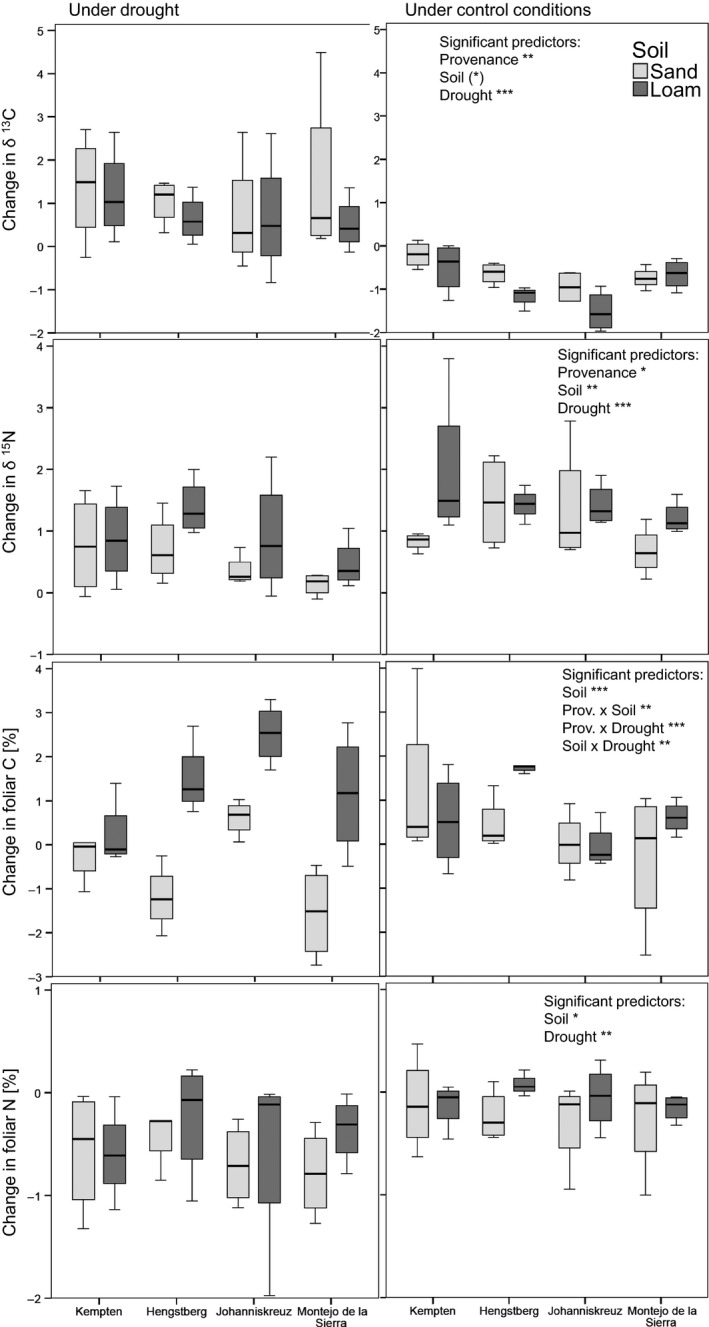
Change of foliar δ^13^C, δ^15^N, C, and N over the drought period between May and June depending on soil quality. Significant variables in the Linear models are summarized in the right‐side graphs for each dependent variable; for detailed statistical results, see Table 3.

Provenance and drought significantly influenced δ^13^C values, soil quality had a marginally significant effect (*p* = .077), and interactions were nonsignificant (Fig. 1, Table 3). According to post hoc comparison, Kempten saplings showed a significantly larger change in δ^13^C as compared to Johanniskreuz saplings indicating a higher level of plant stress for Kempten beeches. Saplings on sandy soil showed a trend toward a larger change in δ^13^C suggesting higher stress than in loamy soil. The magnitude of the drought effect between control and drought‐treated saplings, however, was similar in both soils (Table 4, Fig. 1). δ^13^C value changes during drought were clearly more variable than the values under control conditions (Fig. 1). Regarding control conditions on both soils separately, beeches from Kempten showed higher δ^13^C value changes for both soils compared to Hengstberg and Johanniskreuz (according to post hoc comparison; compare to Fig. 1).

**Table 4:** Median (minimum/maximum) of the change of the values of foliar C, N, δ ^13^C, and δ ^15^N during drought treatment between early May and mid‐June

		Kempten	Hengst‐berg	Johannis‐kreuz	Montejo d. la Sierra	Prov.*F* (*p*)	Drought*F* (*p*)	Prov × Drought*F* (*p*)
Sandy soil								
Control	Foliar Cchange (%)	0.39 ab (0.07/3.98)	0.2 ab (0.02/1.32)	−0.01 a (−0.81/0.92)	0.14 b (−2.51/1.03)	**3.128** * **(.044)**	**9.46** ** **(.005)**	**4.83** ** **(.009)**
Drought		−0.04 (−1.07/0.05)	−1.24 (−2.07/−0.26)	0.68 (0.06/1.02)	−1.52 (−2.73/−0.47)			
Control	Foliar N change (%)	−0.15 (−0.63/0.47)	−0.3 (−0.44/0.1)	−0.12 (−0.95/0.01)	−0.11 (−1/0.19)	0.334 (.801)	**7.5** * **(.011)**	0.3 (.823)
Drought		−0.45 (−1.32/−0.04)	−0.28 (−0.85/−0.28)	−0.71 (−1.12/−0.26)	−0.79 (−1.27/−0.29)			
Control	Foliar δ ^13^C change	−0.19 a (−0.55/0.13)	−0.6 ab (−0.96/−0.41)	−0.96 b (−0.13/−0.62)	−0.76 ab (−1.03/−0.43)	2.75 (.065)	**70.39** *** **(<.001)**	0.758 (.529)
Drought		1.49 (−0.25/2.7)	1.2 (0.32/1.14)	0.31 (−0.45/2.64)	0.66 (0.18/4.49)			
Control	Foliar δ ^15^N change	0.86 (0.63/0.95)	1.46 (0.73/2.22)	0.97 (0.7/2.79)	0.64 (0.22/1.19)	2.275 (.106)	**9.68** ** **(.005)**	0.469 (.706)
Drought		0.75 (−0.06/1.65)	0.61 (0.15/1.45)	0.26 (0.18/0.73)	0.18 (−0.1/0.28)			
Loamy soil								
Control	Foliar C change (%)	0.51 (−0.67/1.81)	1.76 (1.6/1.77)	−0.24 (−0.43/0.71)	0.6 (0.16/1.06)	2.69 (.07)	3.14 (.09)	**4.93** ** **(.009)**
Drought		−0.11 (−0.27/1.4)	1.25 (0.75/2.7)	2.54 (1.7/3.3)	1.17 (−0.49/2.77)			
Control	Foliar N change (%)	−0.49 (1.09/3.79)	0.05 (−0.04/0.21)	−0.04 (−0.44/0.31)	−0.12 (−0.32/−0.05)	1.544 (.23)	3.98 (.058)	0.259 (.854)
Drought		−0.61 (−1.14/−0.04)	−0.71 (−1.05/0.22)	−0.12 (−1.98/−0.02)	−0.31 (−0.79/−0.01)			
Control	Foliar δ ^13^Cchange	−0.36 (−1.26/0)	−1.08 (−1.51/−0.97)	−1.58 (−1.97/−0.93)	−0.63 (−1.08/−0.29)	2.27 (.107)	**62.79** *** **(<.001)**	1.026 (.399)
Drought		1.03 (0.11/2.63)	0.58 (0.05/1.37)	0.48 (−0.84/2.61)	0.41 (−0.13/1.36)			
Control	Foliar δ ^15^N change	1.49 (1.09/3.79)	1.44 (1.11/1.74)	1.32 (1.14/1.9)	1.13 (0.99/1.59)	1.604 (.216)	**8.724** ** **(.007)**	0.442 (.725)
Drought		0.84 (0.05/1.73)	1.28 (0.97/2)	0.75 (−0.06/2.2)	0.35 (0.11/1.04)			

Data were rank‐transformed prior to analyses due to the lack of normality and homogeneity of variances. Significant results are highlighted and marked with asterisks (*** if *p* < .001; ** if *p* < .01; * if *p* < .05). If provenance was at least marginally significant, a Tukey's post hoc test was carried out. Different small letters next to the median indicate significant differences with *p* < .05 between the specific provenances.

#### 3.1.2 Changes in δ^15^N values during drought

Provenance, drought, and soil quality influenced δ^15^N significantly. Montejo de la Sierra saplings showed significantly smaller changes in δ^15^N compared to Hengstberg saplings according to post hoc comparisons (mean difference in ranks: −17.05, *p* = .016). Beech saplings in loamy soil showed larger changes in δ^15^N compared to those grown in sandy soils as did saplings grown under control conditions compared to those subjected to drought. Foliar δ^15^N increased within the period of early May–mid‐June, and this increase was smaller under drought (Table 4). δ^15^N changes were more positive in loamy than in sandy soil. Only Hengstberg saplings under control conditions showed a similar median increase in the δ^15^N values in both soils (Fig. 1).

Including tree height in May as a covariate into the LM to explain foliar δ^15^N in May did not improve the model. The Pearson correlation between the tree height and stem diameter in May with foliar δ^15^N was also not significant (*r* = 0.11, *p* = .38 and *r* = 0.14, *p* = .27, respectively). However, there were significant correlations between the change in tree height and the change in foliar δ^15^N (*r* = 0.37, *p* = .003) and between the change in stem diameter and the change in foliar δ^15^N (*r* = 0.43, *p* < .001) for the period from the beginning of May and mid‐June.

#### 3.1.3 Changes in foliar C and N values during drought

Soil quality had an especially high influence on changes in foliar C content particularly in the drought treatment, with greater changes in C content in loamy soil than in sandy soil. However, interaction terms (soil × provenance and soil × drought) were significant as well which demonstrates that this soil quality effect on foliar C content was not equally strong for all provenances and under control conditions compared to drought conditions (Fig. 1). Drought had a significant negative effect on foliar C on sandy soil (Table 4) and only saplings from Johanniskreuz continued to increase foliar C under drought conditions. In loamy soil, there was a significant interaction with provenance: Kempten and Hengstberg saplings accumulated less foliar C during drought compared to the control whereas saplings from Johanniskreuz and Montejo de la Sierra gained more foliar C under drought compared to the control plants (Table 4, loamy soil). On sandy soil, there was a similar interaction but only saplings from Johanniskreuz accumulated more foliar C under drought conditions compared to control plants, whereas Montejo de la Sierra saplings showed a similar pattern to those from Hengstberg and Kempten. However, saplings from Montejo de la Sierra produced more new leaves between early May and mid‐June compared to plants from Kempten and Johanniskreuz, which was significant for sandy soil and a nonsignificant trend for loamy soil.

Foliar N decreased over the period of early May to mid‐June on sandy soil and in most cases also on loamy soil. The decrease in foliar N was clearly stronger under drought, which was significant on sandy soil but only a trend on loamy soil (Table 4). Kempten saplings showed a strong decrease in foliar N under control and drought conditions, which was similar on both soils. The foliar N of saplings from other provenances underwent little change under control conditions but decreased under drought (Table 4).

### 3.2 Growth parameters after drought

**Figure 2:**
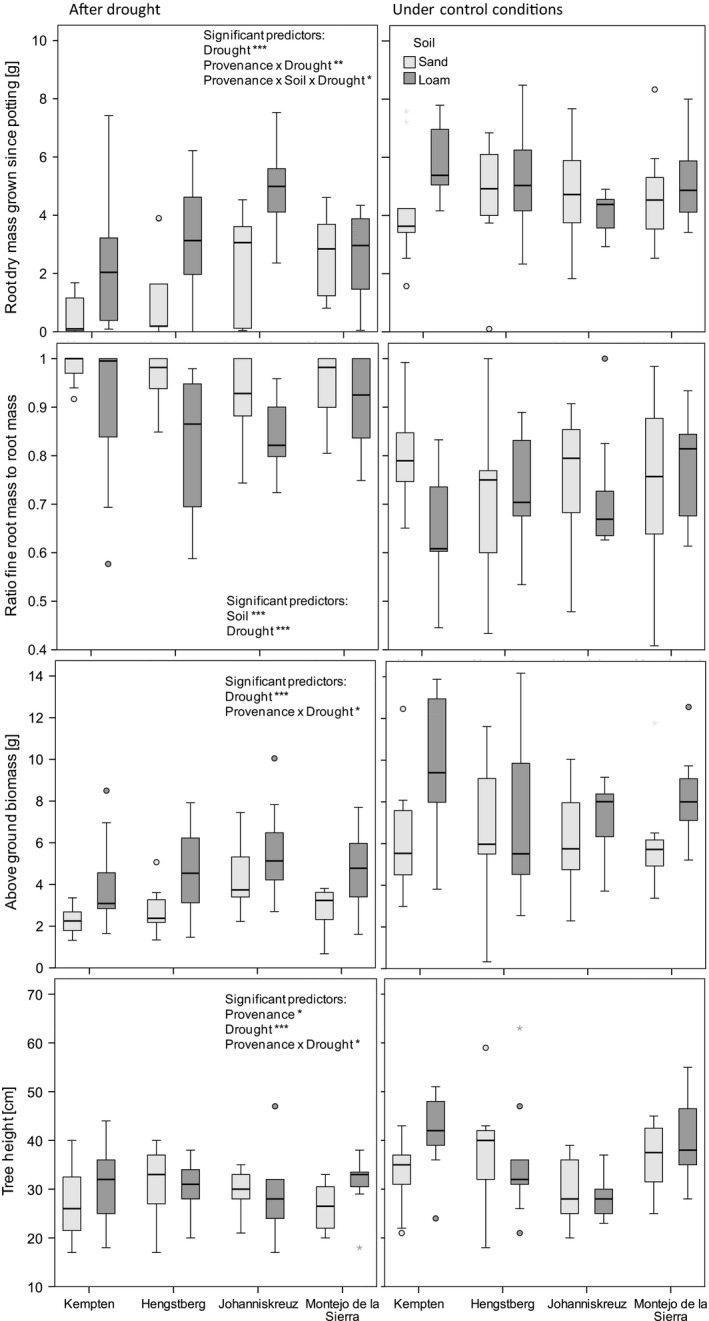
Boxplots of root mass, fine root to root ratio, aboveground dry mass, and tree height on 10th of April after the experiment roughly one1 year after the drought treatment. For detailed Linear model results, see Tables 5 and 6; *p < .05, **p < .01, ***p < .001.

The full models explain roughly 45% of aboveground biomass (*R* ^2^ = .43), root mass (*R* ^2^ = .43) and the relative proportion of fine roots (*R* ^2^ = .46 ) in the spring after the drought treatment (Table 5). The most important influencing factor was drought, which reduced above‐ and belowground biomass dramatically but increased the proportion of fine roots. The latter was also influenced by soil type: The proportion of fine roots relative to total root biomass was greater in sandy soil compared to loamy soil (Table 6). Tree height at the end of the experiment was directly influenced not only by provenance and drought but also by the interactions between provenance × soil and provenance × drought, indicating provenance‐specific responses to soil quality and drought (Table 5). In the full model, saplings from Johanniskreuz reached significantly lower height independent of treatment and soil compared to the other provenances (post hoc comparison). This difference was especially strong in loamy soil and under control conditions. There was a strong negative effect of drought on above‐ and belowground biomass for both soils (Table 6, Fig. 2). For root mass, the magnitude of the difference was clearly higher on sandy soil (median change of 2.53) compared to loamy soil (median change of 1.78). Saplings from Johanniskreuz remained small irrespective of the growing conditions (median height remained stable around 28–30 cm). In loamy soil, saplings from Johanniskreuz were significantly smaller by the end of the experiment than those from Kempten and Montejo de la Sierra (Fig. 2, Table 6). This was in accordance with the lowest tree growth and stem diameter change between early May and mid‐June of Johanniskreuz saplings in loamy soil compared to the other provenances (Table 6). The other provenances performed worse in sandy soil than in loamy soil, resulting in similar final sapling heights and similar changes in tree height and stem diameter change (Table 6).

**Table 5:** Linear model's *F*‐ and *p*‐values on root and shoot biomass, the ratio of fine roots <1 mm to the total root mass and plant height during early spring the year after the treatment including all main effects (provenance, soil, and drought treatment) and all interactions (provenance × soil, provenance × drought, soil × drought, and provenance × soil × drought) as predictors

	Prov*F*/*p*	Soil*F*/*p*	Drought*F*/*p*	P × S*F*/*p*	P × D*F*/*p*	S × D*F*/*p*	P × S × D*F*/*p*	*R*²
Root mass	1/.39	0.01/.93	62.4/**<.001**	1.74/.16	4.15/**.01**	2.52/.12	2.81/**.04**	.43
Fine root/root	1.07/.36	11.59/**.001**	80.24/**<.001**	1.05/.38	0.87/.46	2.65/.11	0.91/.44	.46
Above biomass	1.01/.39	0.1/.75	78.4/**<.001**	0.76/.52	3.19/**.03**	0.78/.38	0.54/.66	.43
Tree height	3.53/**.02**	0.08/.78	17.03/**<.001**	2.66/**.05**	2.96/**.04**	0.06/.81	0.41/.75	.27

*p*‐Values indicating significant differences (*p* < .05) are written in bold.

**Table 6:** Median (minimum/maximum) of the change of the values of tree morphology during drought treatment between early May and mid‐June and of the measures biomass above and below ground 1 year after the treatment

		Kempten	Hengst‐berg	Johannis‐kreuz	Montejo d. la Sierra	Prov.*F* (*p*)	Drought*F* (*p*)	Prov × drought*F* (*p*)
Sandy soil								
Control	Tree height change (cm)	2.5 (1.5/10)	6.5 (−1.5/26.5)	2.5 (1/8)	7.5 (1/17.5)	0.55 (.65)	**34.62** *** **(<.001)**	0.88 (.46)
Drought		3 (−1/4.5)	−0.5 (−2/3.5)	0.5 (−1/2.5)	−0.25 (−1.5/4.5)			
Control	Stem diameter change (mm)	1.75 (1.2/2)	1.85 (0.25/2.45)	1.75 (1.1/2.05)	1.5 (1.1/2.1)	0.98 (.41)	**146.31** *** **(<.001)**	1.53 (.22)
Drought		0.4 (−0.2/1)	0.1 (−0.45/0.9)	0.15 (−0.2/0.9)	0.7 (0.2/1.1)			
Control	Leaf number change	0 a (−6/29)	13 ab (0/50)	2 a (−1/23)	13 b (0/48)	**4.73** ** **(.005)**	**22.44** *** **(<.001)**	1.32 (.277)
Drought		0 (−4/4)	−1 (−6/8)	−1 (−13/4)	2 (−2/14)			
Control	Root mass (g)	3.63 (1.57/7.58)	4.92 (0.1/6.84)	4.72 (1.83/7.67)	4.53 (2.53/8.33)	2.68 (.055)	**51.51** *** **(<.001)**	0.81 (.494)
Drought		0.25 (0.04/1.63)	0.25 (0.1/3.9)	3.06 (0.04/4.53)	2.85 (0.81/4.61)			
Control	Fine roots/all roots	0.79 (0.65/0.99)	0.75 (0.43/1)	0.8 (0.48/0.91)	0.76 (0.41/0.98)	0.773 (.514)	**60.69** *** **(<.001)**	0.377 (.77)
Drought		1 (0.91/1)	0.98 (0.85/1)	0.93 (0.74/1)	0.98 (0.8/1)			
Control	Aboveground biomass (g)	5.53 (3/12.5)	5.97 (0.3/11.6)	5.75 (2.3/10)	5.72 (3.4/11.8)	1.68 (.18)	**56.79** *** **(<.001)**	2.23 (.093)
Drought		2.18 (1.3/3.4)	2.48 (1.7/5.1)	3.74 (2.2/7.5)	3.24 (0.7/3.8)			
Control	Tree height after 1 year (cm)	35 (21/43)	40 (18/59)	28 (20/39)	37.5 (25/45)	1.47 (.23)	**9.11** ** **(.004)**	1.77 (.162)
Drought		28 (21/40)	33.5 (27/40)	30 (21/35)	26.5 (20/33)			
Loamy soil								
Control	Tree height change (cm)	5 ab (−1.5/22.5)	5 a (0/17.5)	1 b (−2/2)	3.5 ab (−5/9.5)	2.69 (.054)	18.16 *** **(<.001)**	2.57 (.062)
Drought		0 (−1.5/4)	1 (−1/3.5)	1 (−1.5/3.5)	0 (−0.5/1.5)			
Control	Stem diameter change (mm)	2.2 a (1.75/2.95)	2.05 a (1.85/2.2)	1.7 b (1.25/2.4)	1.95 ab (1.1/2.5)	**5.22** ** **(.003)**	**239.89** *** **(<.001)**	0.05 (.98)
Drought		0.7 (0.35/1.35)	0.85 (0/1.1)	0.85 (0/1.1)	0.63 (0.3/1.15)			
Control	Leaf number change	3 (−8/24)	3 (−1/25)	3 (−1/14)	5 (−6/38)	2.23 (.094)	**5.95** * **(.018)**	0.72 (.542)
Drought		−2 (−4/0)	1 (−7/13)	1 (−7/13)	0 (−1/13)			
Control	Root mass (g)	5.38 (4.16/7.79)	5.03 (2.33/8.48)	4.38 (2.93/4.9)	4.87 (3.42/8)	0.355 (.785)	**17.69** *** **(<.001)**	**5.557** ** **(.002)**
Drought		2.04 (0.09/7.42)	3.39 (0.96/6.22)	4.99 (2.36/7.53)	2.96 (0.05/4.34)			
Control	Fine roots/all roots	0.61 (0.45/0.83)	0.7 (0.53/0.89)	0.67 (0.63/1)	0.81 (0.61/0.93)	1.27 (.292)	**25.09** *** **(<.001)**	1.38 (.256)
Drought		1 (0.58/1)	0.87 (0.59/0.98)	0.82 (0.72/0.96)	0.93 (0.75/1)			
Control	Aboveground biomass (g)	9.5 (3.8/13.9)	5.51 (2.6/14.2)	8.01 (3.7/9.2)	8 (5.2/12.6)	0.31 (.819)	**27.4** *** **(<.001)**	1.62 (.194)
Drought		3.09 (1.7/8.5)	4.7 (1.5/7.9)	5.13 (2.7/10.1)	4.78 (1.6/7.7)			
Control	Tree height after 1 year (cm)	42 a (24/51)	32 ab (21/63)	28 b (23/37)	38 a (28/55)	**4.87** ** **(.004)**	**7.91** ** **(.007)**	1.6 (.198)
Drought		32 (18/44)	30 (20/38)	28 (17/47)	33 (18/38)			

Data were rank‐transformed prior to analyses due to the lack of normality and homogeneity of variances. Significant results are highlighted and marked with asterisks (*** if *p* < .001; ** if *p* < .01; * if *p* < .05). If provenance was at least marginally significant, a Tukey's post hoc test was carried out. Different small letters next to the median indicate significant differences with *p* < .05 between the specific provenances.

### 3.3 Leaf and growth parameters (absolute values) at the beginning and the end of the experiment, survival

Saplings from different provenances performed different during their first year of growth, before being potted for our experiment: In March, directly after planting, saplings from Johanniskreuz were taller than all the others (Table S1; significant and marginally significant differences according to post hoc comparison). The discrepancy vanished during the growing season: In May, no more significant differences between the provenances were found but the interaction term provenance × soil became significant: Whereas saplings from Hengstberg and Johanniskreuz were of equal height in both soils, saplings from Kempten and Montejo de la Sierra tended to grow taller in loamy soil than in sandy soil. Stem diameter and the number of leaves in May were significantly larger in Johanniskreuz saplings than in any other provenance (Table S1, according to post hoc comparison). Foliar C, N, and isotope signatures in May before the drought treatment had started are illustrated in Fig. S2. Although there were no significant differences in foliar C content among saplings from different provenances, C content and N content in three of the four provenances were lower in loamy soil than on sandy soil. Foliar N, δ^13^C, and δ^15^N were different between provenances before the drought (Fig. S2). Saplings from Johanniskreuz and Montejo de la Sierra had higher foliar N values compared to saplings from Hengstberg and Kempten. Saplings from Montejo de la Sierra had higher δ^15^N values than those from Hengstberg and saplings from Johanniskreuz had higher δ^13^C than saplings from Hengstberg.

By April, roughly 1 year after the drought treatment, two of the nine beech saplings from Kempten grown in sandy soil under drought conditions had died, as had one drought‐treated beech sapling from Hengstberg in sandy and in loamy soil. All the others had survived after 1 year, although some were in a very poor state.

## 4 Discussion

### 4.1 δ^13^C values and total C (%)

The drought led to expected positive changes in the δ^13^C values indicating water stress and the partial and temporal closure of the stomata. We expected loamy soil to buffer the drought impact on the plants more than sandy soil due to the better water retention capacity. However, δ^13^C values were only marginally different between the soils (higher in sandy soil compared to loamy soil), indicating a trend toward higher stress and more frequent stomata closure in sandy soil compared to loamy soil. Under control conditions, discrimination of ^13^C took place in expected magnitudes over the period between May and mid‐June (compare to Damesin, Rambal, & Joffre, 1998 or to Fotelli et al., 2003). Discrimination of ^13^C was less pronounced on sandy soil (median change—0.73) than in loamy soil (median change—0.86), which suggests that even under average weather conditions stomata were kept closed more frequently on sandy soil than in loamy soil.

Beech saplings growing in sandy soil severely reduced foliar C during drought except for one provenance: Saplings from Johanniskreuz showed foliar C enrichment during drought in both soils. It is possible that the drought was severe enough in the sandy soil to start the process of C‐starvation in saplings from the other provenances. However, the study of starch pools of the whole plant would be necessary to provide evidence of limiting C resources for plant metabolism and survival (McDowell & Sevanto, 2010). In contrast to sandy soil, foliar C kept increasing between May to June for all provenances in loamy soil. Accordingly, stomatal closure must have been more pronounced for plants growing in sandy soil compared to loamy soil. In contrast to the drought reaction, strong foliar C loss did not occur under control conditions. Here, C values remained more or less stable over time, which is in accordance with other studies (Nahm et al., 2007; Wang, Xu, & Schjoerring, 2011). However, the foliar C loss found during drought is not consistent to other studies. Peuke and Rennenberg (2004) measured no change in total leaf carbon during drought in beech seedlings of 11 different provenances. In that study, drought was controlled at 20% volumetric water content, which was clearly less pronounced than in the present study, where values below 10% were reached. Additionally, Peuke and Rennenberg (2004) used a well‐fertilized mixture adding commercial potting soil probably characterized by good water holding capacity. Consequently, we assume that only severe drought initiates foliar C reduction as found on sandy soil in our experiment.

### 4.2 δ^15^N values and total N [%]

Drought had a significant negative effect on δ^15^N values. This was not expected, as other studies found no such response (Peuke et al., 2006). However, δ^15^N should be correlated with plant size as the discrimination process of ^15^N due to transport within the plant takes place over longer time or distances if a plant is larger (Peuke et al., 2006). In our study, tree height was significantly reduced by drought compared to control conditions, and the change in δ^15^N between early May and mid‐June was clearly correlated with the growth of the plants within the same period of time over all treatments and provenances. There was no significant difference of δ^15^N value changes over the drought period between sandy and loamy soils. However, δ^15^N values were higher in saplings growing in loamy soil than in sandy soil. This trend shows that the potential growth reduction and consecutive reduction in ^15^N discrimination due to drought was overlaid by a similar effect due to limited growth in sandy soil. As the sandy soil was poorer in N compared to the loamy soil, the fractionation during N uptake was probably also lower on sandy soil (Craine et al., 2015).

Foliar N (%) content of the leaves in May was comparable to other measurements, for example, those undertaken by Wang et al. (2011). Values declined over the duration of the experiment in nearly all treatments, which has been also described by Geßler et al. (2007). An explanation could be that leaf growth and chlorophyll synthesis is terminated by the month of May leading to a reduction in soluble N content after spring (Nahm et al., 2006). In our study, the reduction in foliar N after the drought in loamy soil was not as pronounced as in sandy soil. In particular, the control plants in loamy soil showed remarkably stable foliar N values. Accordingly, it is unlikely that in our study the termination of leaf growth after spring was responsible for the foliar N reduction found especially in sandy soil and after drought but might have its origin in hampered N uptake due to the water deficit. Foliar nitrogen may also remain stable over summer (Wang et al., 2011) under good conditions. The sandy soil was also the nutrient poor soil in our experiment for three reasons: The sandy compartment included comparably few nutrients (Table 2), the stronger water deficit in the sandy soil compared to the loamy soil leads to nutrient shortage as water is necessary for nutrient uptake and third, and the high magnesium (Table 2) and probably also high calcium content in the sand could fix phosphorus as calcium phosphate and makes it unreachable for the plants (Schlesinger & Bernhardt, 2013). The latter is not specific to all sandy soils, as calcium is often washed out from the sand. However, acidic sand would could lead to even worse nutrient conditions as nutrient availability is generally better under higher than under lower soil pH (Schlesinger & Bernhardt, 2013). The possible lack of available P on sand could be—next to the different N content—a second crucial factor determining photosynthesis and water use efficiency effects on sandy soil compared to loamy soil (Minotta & Pinzauti, 1996; Peuke & Rennenberg, 2004; Schlesinger & Bernhardt, 2013).

Although the beech saplings were of different sizes at the beginning of the experiment, the initial foliar δ^15^N values were not correlated with growth during the first year. This may again be explained by the time shift and nutrient transport processes between tree growth during the first year and leaf production in the following spring (Geßler et al., 2007; Nahm et al., 2006). The trees that grew especially well during their first year (saplings from Johanniskreuz and Montejo de la Sierra) did not grow well between March and the beginning of May in their second year. Both were significantly late (about 5 days) in their phenology as compared to saplings from Kempten (C. Buhk, unpublished data). In addition, beech saplings from Johanniskreuz and Montejo de la Sierra had significantly higher foliar N content than beeches from Kempten and Hengstberg. As high foliar N content is correlated with chlorophyll content and to the CO_2_ assimilation rate (Evans, 1989), photosynthesis and therefore water use efficiency might be more effective (Schlesinger & Bernhardt, 2013) for saplings from Johanniskreuz and Montejo de la Sierra, which allows them to close the stomata regularly without risking C‐starvation. According to Peuke and Rennenberg (2004), leaf nitrogen concentration remained stable under drought. In their study, N was also highly dependent on provenance, which is partly in accordance with our study. Provenances showed significantly different foliar N values during the start of the experiment in May but changes in foliar N during the experiment were not provenance specific but influenced by drought—especially on sandy soil (Table 4).

### 4.3 Fine root to root ratio

Although overall root mass was reduced in the drought‐treated plants and in sandy soil in the year after the experiment (with the exception of Johanniskreuz saplings in loamy soil after drought), the ratio of fine roots to the total root biomass was clearly higher after drought and in sandy soil compared to loamy soil. This could reflect that nutrient and water uptake in the sandy soil and after drought depends mainly on fine roots. However, it may also be the result of the droughted plants forming new roots during late summer and autumn when growing conditions were more favorable.

### 4.4 Provenance‐specific behavior

Along with the climatic conditions at their geographic origin, some populations seem to be more adapted to drought than others. Drought probability (and other environmental conditions) at the geographic origin of plants may partly determine their drought response. Hence, the beech saplings retrieved from Spain (Montejo de al Sierra; see drought index Table 1) should reveal clearer drought adaption than the other provenances.

#### 4.4.1 Beech saplings from Montejo de la Sierra

Leaf damage, mortality, and growth followed the expectations that beech saplings from Montejo de la Sierra were pre‐adapted to drought (Thiel et al., 2014), but this was not apparent in the δ^13^C values. There was no indication that beech saplings from Montejo de la Sierra had to close their stomata less often than the others—especially in sandy soil. Discrimination of ^13^C under drought was stronger compared to Johanniskreuz saplings in sandy soil but was otherwise the lowest of all provenances in loamy soil. Apparently, other adaptation strategies might be found. Aranda, Gil, and Pardos (2000) studied beeches (30 year saplings) from Montejo de la Sierra concerning their water potential and stomatal conductance and photosynthesis in the field. They showed that the stomata were closed during the hottest time of the day. However, the minimum midday water potential reached in the study of the Spanish beech trees is lower (−2 to −2.4 MPa) than the threshold documented for beech xylem embolism of −1.9 mentioned by Aranda et al. (2000) and Hacke and Sauter (1995). This indicates that the Spanish beech saplings may be protected by more stable xylem cells compared to those studied by Hacke and Sauter (1995) preventing cavitations and consecutively hydraulic failure (McDowell et al., 2008). The Spanish beech saplings may not be strictly isohydric (trying to keep the water potential stable by closing the stomata) but show also anisohydric behavior, allowing very strong negative water potential as the xylem is more resistant to embolism (Klein, 2014). This may prevent them from carbon starvation during the regularly long lasting drought events common in Montejo de la Sierra (Aranda et al., 2005; McDowell et al., 2008). However, foliar carbon loss in sandy soil was strong for the Spanish trees; the aboveground dry mass of the drought‐treated plants was clearly reduced 1 year after the treatment, indicating that growth was strongly negatively influenced by the drought. Similarly strong drought effects on growth has also been found in beech trees from xeric sites in Sicily, although their stomata had been kept open longer during the drought compared to Italian beech trees from a mesic site (Tognetti et al., 1995). Root mass and the proportion of fine roots of the Spanish saplings remained comparatively stable between the treatments and soils compared to the other provenances. Although their growth was clearly hampered, all Spanish beech saplings survived the severe drought event and profited from good water supply under control conditions especially in loamy soil by very healthy growth. This reaction indicates high plasticity in the growth response of Montejo de la Sierra beech saplings depending on the conditions. In line with this observation, southern provenances were found to show a much stronger positive response to increase in soil water content than northern provenances (Nielsen & Jørgensen, 2003).

#### 4.4.2 Beech saplings from Kempten

Provenance Kempten has a SHMI of only 26 (compared to 80 in Montejo de la Sierra) due to cool and humid summers. Despite this, according to our δ^13^C values, saplings did not appear to have closed the stomata more often during drought than other provenances, although a trend was visible. However, trees from Kempten showed the highest level of leaf injury and higher mortality (Thiel et al., 2014), so they were obviously more stressed than saplings from the other provenances, but this was not indicated by higher δ^13^C values in mid‐June in our study. To find an explanation, we point to the high variation in δ^13^C value changes among individuals within all provenances after drought. This could be the result of three overlying processes: (1) Over time ^13^C is discriminated during photosynthesis, leading to a natural downwards trend of δ^13^C (Fotelli et al., 2003). (2) Discrimination is reduced if stomata are kept closed (Fotelli et al., 2003), and (3) δ^13^C remains unchanged if photosynthesis collapses and no further C is incorporated into the leaves; if this took place at different times for each individual and leaf, this could explain the high variation in the drought data. In contrast to the other provenances, foliar C content in trees from Kempten remained largely unchanged which could indicate a collapse in photosynthesis and hydraulic and symplastic failure (McDowell et al., 2008) at an early point in time. Therefore, leaf mortality (Thiel et al., 2014) seems a better indicator to observe drought response of such a severe drought than δ^13^C in this study. Kempten beech saplings seemed to grow very well under good site conditions (in loamy soil under control conditions), but they were not adapted to the drought and probably suffered from embolisms: Two replicates in sandy soil died under drought during the experiment and two more plants showed only very low vitality in April of the following year, when the dry mass of the trees grown in sandy soil and treated by drought was much lower compared to the control in loamy soil.

#### 4.4.3 Beech saplings from Hengstberg

According to Thiel et al. (2014), beech saplings from Hengstberg take an intermediate position between Kempten and Montejo de la Sierra saplings in terms of leaf injuries, mean diameter reduction, and mortality in response to drought. However, here we demonstrate that the final aboveground biomass 1 year after the treatment tended to be lower under good conditions (control in loamy soil) than all the other provenances. Saplings from Hengstberg probably lacked the plasticity of the Kempten beeches but were able to cope with the extreme drought fairly well. This is in line with observations from another experiment studying beech trees from Hengstberg (Harter et al., 2015), in which control and drought treatments lead to very similar height increment. The different soil types in our study, however, showed the limits of saplings of this provenance, as dry mass remained especially low under drought in sandy soil. This might be directly linked to the completely different soil texture in its home range, which is clay loam.

#### 4.4.4 Beech saplings from Johanniskreuz

The growth of Johanniskreuz beech saplings, the second intermediate provenance between Montejo de la Sierra and Kempten, was not very good during the experiment, but leaf injuries were low (Thiel et al., 2014) and C concentrations in the leaves were highest. At the beginning of the experiment in March, beech saplings from Johanniskreuz were taller, thicker (stem diameter), and had clearly more leaves than beeches from the other provenances. Saplings from Johanniskreuz were also not similarly stressed in sandy soil compared to saplings from the other provenances, as they clearly showed the lowest rise in δ^13^C values in sandy soil, which also correlated well with the leaf carbon pattern found: Foliar C rose significantly more during drought on both soils compared to all other provenances. Foliar N was as high as in the Spanish beech saplings, and root mass in sandy soil after drought was high in comparison with plants from Hengstberg and Kempten in a similar range as the Spanish beech saplings. These parameters seemed to indicate high growth potential. The beech saplings from Johanniskreuz originated from sandy substrates with low utilizable field capacity within the root zone of only about 90 mm (Ehses, 2013). Consequently, they were the only beech saplings that grew just as well in sandy soil as in loamy soil—maybe as a consequence of adaptation to unfavorable soil conditions (Pluess & Weber, 2012). The elevated foliar δ^13^C values at the beginning of the experiment in May are difficult to interpret: They could indicate drought stress and stomatal closure, but the plants grew especially well during their first season, which would exclude the possibility of limited photosynthesis rates due to stomata closure. This contradiction was already noted by Tognetti et al. (1995), who found that growth and photosynthesis in *F. sylvatica* are poorly related. As foliar C value and the number of leaves were high in Johanniskreuz beeches, we exclude the explanation that photosynthesis was limited due to stomatal closure resulting in the low foliar δ^13^C values. Instead, we propose that Johanniskreuz beech saplings retranslocated carbon within the plant leading to lower δ^13^C discrimination within the plant during their first year of growth. Indeed, isotope composition in leaves in spring mirror the growing conditions during the former season, but it is clearly modified during transport processes from leaves to storage organs in autumn and back to the buds in spring (Nahm et al., 2006, 2007; Peuke et al., 2006).

## 5 Conclusion

We conclude that soil quality has a strong effect on the drought response of beech saplings. Sandy soil aggravated the drought for all provenances, and the effect of texture is certainly coupled with lower nutrient availability in sandy soil, although we cannot separate these effects. Saplings from Johanniskreuz were able to cope with sandy conditions best, probably due to local adaptation to the sandy soils in their home range. δ^13^C values turned out to be a bad indicator of beech drought stress when the drought event is severe and different mechanisms of discrimination due to stomatal closure and cessation of photosynthesis might overlie each other. As most studies do not let the plants die during their experiment, there is a lack of knowledge of the processes that lead to death as most studies “…confuse stress responses with mortality mechanisms” as stated by McDowell and Sevanto (2010). In our study, we find stress responses in loamy soil for three of the four provenances. Strategies to cope with drought as well as response plasticity seem to differ strongly among provenances. Local adaptation includes more environmental factors than just climate. Here, we show that soil quality is clearly another relevant factor to be included into species distribution models. Rough estimates based on soil characteristics, such as those generated from the European Soil Database, might not be detailed enough (Casalegno, Amatulli, Bastrup‐Birk, Durrant, & Pekkarinen, 2011). Provenance‐specific reactions relevant to climate change research should incorporate not only adaptation to climate but also adaptation to soil quality. For practitioners in forestry and conservation, this issue is an additional relevant factor to be taken into account, for example, for reforestation programs (Kreyling et al., 2011).

## Funding Information

Bavarian State Ministry of the Environment and Public Health (Grant/Award Number: ZKL01Abt7_18456).

## Conflict of Interest

None declared.
