# Supplementary material for: On the influence of provenance to soil quality enhanced stress reaction of young beech trees to summer drought
Source: Ecol Evol. 2016 Oct 21;6(22):8276–90. doi: 10.1002/ece3.2472 (PMC5108277; doi:10.1002/ece3.2472)
Supplement: Supplementary file 1 [file ECE3-6-8276-s001.ppt]

## Slide 1
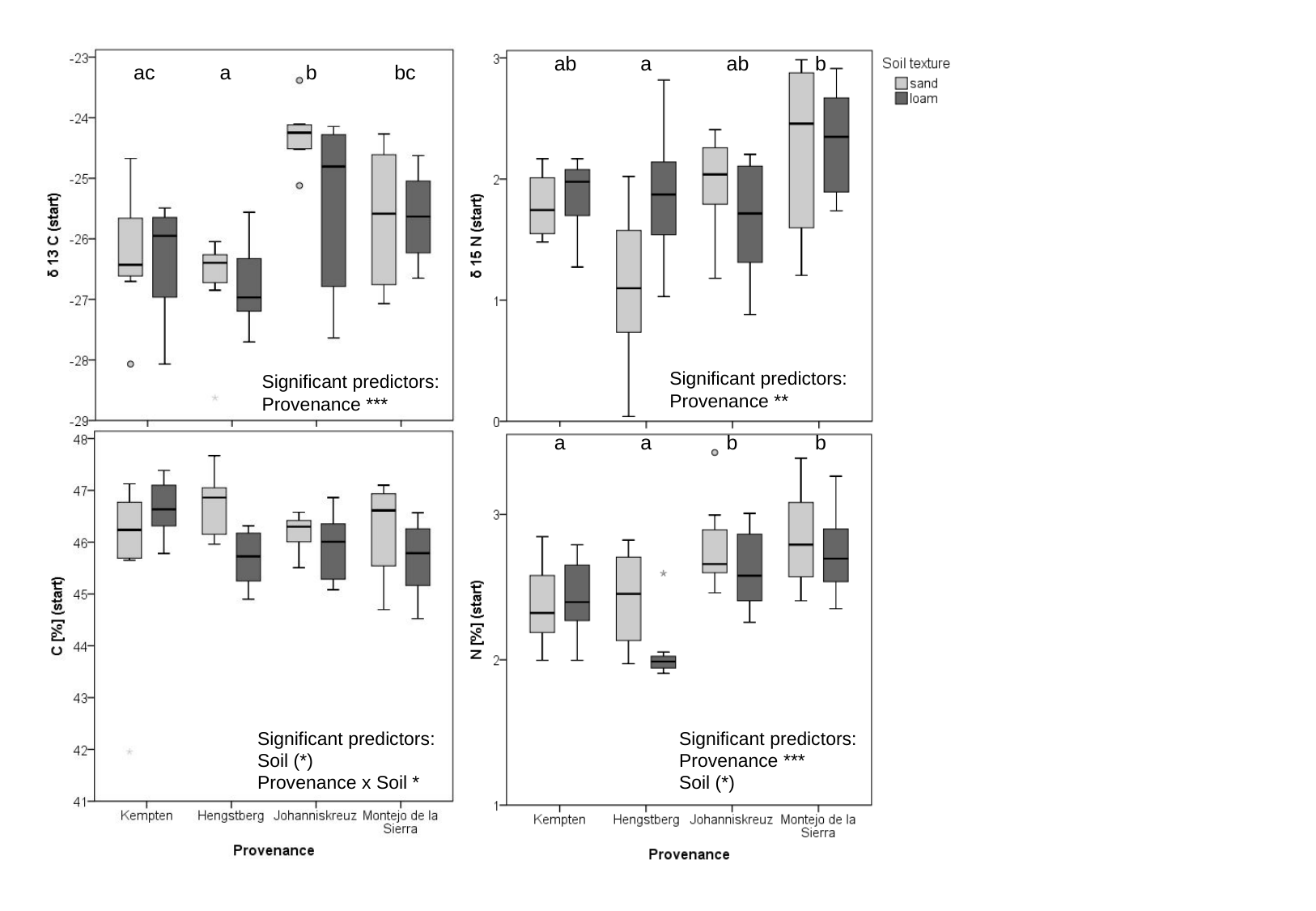

ab
a
ab
b
ac
a
b
bc
Significant predictors:
Provenance ***
Significant predictors:
Provenance **
a
a
b
b
Significant predictors:
Soil (*)
Provenance x Soil *
Significant predictors:
Provenance ***
Soil (*)
