# Supplementary material for: On the influence of provenance to soil quality enhanced stress reaction of young beech trees to summer drought
Source: Ecol Evol. 2016 Oct 21;6(22):8276–90. doi: 10.1002/ece3.2472 (PMC5108277; doi:10.1002/ece3.2472)
Supplement: Supplementary file 2 [file ECE3-6-8276-s002.docx]

Appendix

Table S - 1: Median (Minimum/Maximum) of tree morphology, foliar C, N, δ ^13^C and δ ^15^N measured at the start of the experiment in sandy soil and in loamy soil before the drought treatment. Beech saplings were planted into the mineral soil in March. Initial tree size resulting from last year’s growth is added as tree height (March). All other data result from measurements before the drought treatment that started in early May. Data were rank transformed prior to analyses as data were neither normally distributed nor homoscedastic. Significant results are highlighted and marked with asterisks (*** if p< 0.001; ** if p< 0.01; * if p< 0.05).

|  | Sandy soil | | | | Loamy soil | | | | Prov. | Soil | Prov * Soil |
| --- | --- | --- | --- | --- | --- | --- | --- | --- | --- | --- | --- |
| Provenance | Kempten | Hengstberg | Johanniskreuz | Montejo de la Sierra | Kempten | Hengstberg | Johanniskreuz | Montejo de la Sierra | F  (p) | F  (p) | F  (p) |
| Tree height (March) [cm] | 19.5  (5/28) | 18.5  (13/33) | 25.5  (17/36) | 19.25  (10/28) | 22.5  (10/36) | 19  (9/31) | 24.75  (14/35) | 23.25  (11/34) | **4.77 ****  **(0.003)** | 0.31  (0.578) | 2.66  (0.051) |
| Tree height  (May) [cm] | 27.75  (17/36) | 27.75  (19/40.5) | 28.5  (20/36) | 27.75  (15/32.5) | 30  (19/46,5) | 26  (17.5/35) | 28  (18/36) | 31  (17,5/37) | 0.13  (0.942) | 1.28  (0.26) | **3.17 ***  **(0.03)** |
| Stem diameter (May) [mm] | 4.55  (3/6) | 4.55  (2/6) | 5.13  (4/6) | 4.35  (2/5) | 4.75  (4/7) | 4.13  (3/6) | 5.2  (4/6) | 4.9  (4/6) | **8.16 *****  **(<0.001)** | 0.98  (0.32) | **2.74 ***  **(0.046)** |
| Leaf number  (May) | 27  (14/88) | 39  (8/61) | 56.5  (27/86) | 28  (14/52) | 41.5  (11/97) | 39  (17/66) | 49  (27/82) | 45  (23/61) | **12.03 *****  **(<0.001)** | **4.36 ***  **(0.04)** | **2.88 ***  **(0.04)** |
| Foliar C  (May) [%] | 46.23  (42.0/47.1) | 46.86  (46.0/47.7) | 46.29  (45.5/46.6) | 46.61  (44.7/47.1) | 46.6  (45.8/47.4) | 45.7  (44.9/46.3) | 46  (45.1/64.9) | 45.8  (44.5/46.6) | 1.25  (0.3) | **4.02 ***  **(0.05)** | **3.88 ***  **(0.014)** |
| Foliar N  (May) [%] | 2.32  (2/2.85) | 2.45  (1.97/2.82) | 2.66  (2.46/3.43) | 2.79  (2.41/3.39) | 2.4  (2/2.79) | 1.99  (1.91/2.6) | 2.58  (2.26/3.01) | 2.7  (2.35/3.26) | **10.41 *****  **(<0.001)** | 3.07  (0.085) | 1.175  (0.328) |
| Foliar δ ^13^C  (May) | -26.4  (-28.1/-24.7) | -26.4  (-28.6/-26.1) | -24.3  (-25.1/-23.4) | -25.6  (-27.1/-24.3) | -26  (-28.1/-25.5) | -27  (-27.7/-25.6) | -24.8  (-27.6/-24.2) | -25.6  (-26.7/-24.6) | **10. 5 *****  **(<0.001)** | 1.82  (0.183) | 1.47  (0.233) |
| Foliar δ ^15^N  (May) | 1.75  (1.48/2.17) | 1.10  (0.04/2.02) | 2.04  (1.18/2.41) | 2.46  (1.2/2.99) | 1.98  (1.27/2.17) | 1.87  (1.03/2.82) | 1.72  (0.88/2.2) | 2.35  (1.74/2.91) | **5.29 ****  **(0.003)** | 1.337  (0.253) | 2.125  (0.108) |

Figure legends

Figure S – 2: Boxplots of foliar C, N, δ^13^C and δ^15^N at the start of the experiment before the drought. Data were rank transformed prior to analyses due to the lack of normality and homogeneity of variances. Significant predictors are followed by asterisks (*** if p< 0.001; ** if p< 0.01; * if p< 0.05; (*) if p< 0.1). If provenance was significant, a Tukey post-hoc test was carried out. Different small letters indicate significant differences with p< 0.05 between the specific provenances. Detailed LM results see Table S – 1.
